# Studies on the Influence of Compaction Parameters on the Mechanical Properties of Oak Sawdust Briquettes

**DOI:** 10.3390/ma19010119

**Published:** 2025-12-29

**Authors:** Dominik Wilczyński, Krzysztof Talaśka, Krzysztof Wałęsa, Dominik Wojtkowiak, Łukasz Warguła, Tomasz Domański, Marcin Kubiak, Zbigniew Saternus, Andrzej Kołodziej, Karol Konecki, Maciej Szulc

**Affiliations:** 1 Institute of Machine Design, Faculty of Mechanical Engineering, Poznan University of Technology, 61-138 Poznań, Poland; krzysztof.talaska@put.poznan.pl (K.T.); krzysztof.walesa@put.poznan.pl (K.W.); dominik.wojtkowiak@put.poznan.pl (D.W.); lukasz.wargula@put.poznan.pl (Ł.W.); 2 Department of Mechanics and Basics of Machine Design, Faculty of Mechanical Engineering, Czestochowa University of Technology, 42-201 Częstochowa, Poland; tomasz.domanski@pcz.pl (T.D.); marcin.kubiak@pcz.pl (M.K.); zbigniew.saternus@pcz.pl (Z.S.); 3 Department of Mechanical Engineering, Polytechnic Faculty, University of Kalisz, 62-800 Kalisz, Poland; a.kolodziej@uniwersytetkaliski.edu.pl (A.K.); k.konecki@uniwersytetkaliski.edu.pl (K.K.); 4 MSprojekt plus Sp. z o.o., 64-330 Opalenica, Poland; m.szulc@msprojektplus.pl

**Keywords:** compaction process, sieve process, oak sawdust, ANOVA, briquetting, pelletizing

## Abstract

The paper presents research on the compaction process of oak sawdust as a proposal for the management of post-production waste. The variable input parameters whose influence was studied were the particle size of the sawdust, the compaction force, the temperature of the compaction process, and the moisture content of the sawdust. The results obtained were used to determine the density of the briquette and the value of its Young’s modulus obtained from each test sample. The interaction between the input parameters as variables in the tests and the determined values of density and Young’s modulus was analyzed using ANOVA. The highest density value was recorded for the lowest particle size, the highest compaction force and compaction temperature, and a moisture content of 9%. The highest Young’s modulus *E* value was recorded for a moisture content of 9%, a compaction force of 25 kN, a temperature of 25 °C, and a particle size of *S* < 1 mm. Variance analysis enabled the optimal selection of compaction process parameters, where the main criterion in general terms was to minimize the energy consumption of the compaction process. The best mechanical properties of the briquette can be obtained for process settings of *F* = 5 kN, *M* = 20%, *T* = 25 °C, *S* = 2.5–5 mm.

## 1. Introduction

Energy production methods are currently a major focus of scientific interest. This is due, among other things, to the globally growing demand for energy. There are many reasons for this phenomenon, but the most obvious ones are: the increasing population (it is estimated that the population could reach around 10 billion by 2050) and the economic development of societies—and the associated technological development (in the last 25 years, global average GDP growth has exceeded 2.5% annually, excluding individual years of economic crisis). These factors contribute to the increasing global consumption of energy (including electricity) in everyday life. It has been estimated that between 2013 and 2023, global energy demand grew by an average of around 1.3% per year, and in 2024, this figure reached as high as 2.2%. It is forecast that by 2050, the increase in energy demand will be even higher [1,2].

Thermal and electrical energy obtained from fossil fuels accounts for approximately 85% of all known energy sources globally. The remaining demand is met by renewable sources, including biomass. It is estimated that the share of renewable energy will increase significantly over the next 25 years, with a forecast of at least 30% by around 2050. This is mainly due to the growing environmental awareness of societies, as well as the constantly rising costs of obtaining energy from fossil fuels [3,4].

Currently, renewable energy sources include geothermal power plants, solar devices, hydroelectric and wind power plants, as well as biomass. Biomass is most often directly combusted to obtain thermal energy or undergoes other chemical processes that allow fuel to be obtained from it. Biomass currently accounts for a significant percentage of renewable energy sources. It is estimated that its share in renewable energy sources exceeds 40% in the European Union and reaches as much as 70% in Poland [5,6]. In some parts of the world (usually in less developed regions), biomass has an almost 100% market share among methods of generating thermal energy [7]. Globally, this share is growing year on year, which proves that this renewable energy source is important in the entire energy system. Therefore, research conducted in this area can be considered important and the subject matter relevant.

There are different types of biomass, depending on its source. In addition to biomass of agricultural origin (plant residues and food processing waste), aquatic biomass (algae and aquatic plants used for biofuel production) and waste biomass (industrial and municipal waste), one of the most popular types is wood biomass. This group most often includes biomass from forestry waste (waste from wood processing) and biomass from special energy crops (e.g., willow and poplar) [8,9].

The use of wood biomass, which is waste from wood processing, is in line with current trends in the development of renewable energy sources, while also being one of the ways of managing waste. This type of biomass, taking into account particle size, can be divided into three main groups: wood powder (particles smaller than 1 mm, most often a by-product of wood grinding and milling processes), sawdust (particles ranging from 1 mm to 5 mm in size, which are a by-product of cutting and milling processes) and wood chips (with particle sizes up to 30 mm, which are a by-product of cutting and planning processes). In order to obtain thermal energy from the combustion or gasification of these types of fuels, they are compressed into briquettes or pellets, which improves the technological processes involved in their further processing (including transport and storage) and has a positive effect on the efficiency of the technological process of energy recovery (including combustion or gasification) [10,11].

Many researchers have already undertaken work related to the study of biomass compaction for the purpose of obtaining briquettes and pellets used for energy purposes. The most popular objective of research into this technological process is to determine the impact of process parameters on its course and the quality of the product obtained, in the form of pellets or briquettes. The most frequently studied variable parameters of this process, affecting the compaction process and the properties of the obtained product, are: compaction pressure, moisture content of the compacted material, type of compacted material, process temperature, degree of particle fragmentation before compaction, use of bonding additives, mixing of different types of raw materials in different proportions [12,13,14,15,16,17,18]. Many researchers also analyze the chemical composition of the raw material undergoing briquetting or pelletisation. According to the conclusions of their work, the content of lignin, cellulose and hemicellulose also significantly affects the properties of the resulting product [19,20,21,22,23]. Scientists are also studying the design of equipment used to carry out the biomass compaction process and its impact on the physical properties of the biofuel obtained [24,25,26].

Taking into account studies of specific parameters of the wood biomass compaction process, its course and the characteristics of the obtained biofuel, there are a number of works in which researchers present conclusions regarding the correlation between various parameters of both the input material and the process, and the physical properties of the product.

Adam et al. studied the compaction process of a mixture of spruce and pine sawdust. In their paper, they presented the results of experimental piston compaction tests of this raw material, with a maximum compaction pressure of 200 MPa. At the same time, the authors performed numerical calculations to determine the stress distribution in the briquette during the compaction process, based on an analysis of the distribution of internal forces during its formation. The conclusions from the research indicate a significant influence of friction between the briquette and the compaction sleeve on the stress distribution in the briquette, as well as a large influence of the holding time of the maximum compaction stress on the compaction of the material [27]. This work is a very valuable source of information on the phenomena occurring in the process of obtaining a specific density distribution during the compaction of this type of mixture.

Kamdem, Lemaire and Nikiema conducted research on the compression of wood sawdust, miscanthus grass and wheat straw. In this research, the variable input parameters were: the size of the fractions of individual briquette components, the proportion of individual types of biomass and the compression force. The parameters tested were: density, impact resistance, water resistance and parameters related to the combustion of the briquette obtained (calorific value, ignition time, combustion time and ash residue after combustion). It was shown that all input variables have a significant impact on the tested properties of the briquette. A particularly significant influence was demonstrated in the variability of the composition of the compacted mixture on the parameters related to its combustion [28]. This work is a valuable source of information for researchers studying the combustion process of different types of mixtures, especially in terms of combustion purity and the possibility of obtaining energy from mixtures of different types of biofuels.

Molenda et al. conducted experimental research on the compaction of sawdust from various tree species growing in Eastern Europe: pine, willow, oak, poplar, birch and beech. The researchers determined the effect of sawdust moisture content (2 values tested) and compaction pressure (2 values tested) on: pellet height, density, strength, combustion heat and ash residue. The most important conclusions from their research are that, among the plant species mentioned, oak sawdust obtained the highest mechanical strength parameters after compaction. In addition, it was pointed out that an increase in compaction pressure has a positive effect on pellet strength, while an increase in moisture content has the opposite effect. Despite its excellent mechanical properties and low ash content after combustion, oak pellets did not exhibit the highest combustion heat [29]. This work is a valuable source of information, especially in terms of comparing different types of wood from which sawdust was obtained, in terms of their mechanical strength after compaction.

Garrido, Conesa and Garcia conducted research on the compaction of sawdust and date palm trunks mixed with plastic waste from electronic devices and cars, without the use of an additional bonding agent. The briquetting process for these mixtures was carried out at different component ratios, compaction pressures and temperatures. The conclusions of this research indicate that increasing the briquetting temperature of the tested mixtures has a clearly positive effect on the density and durability of the briquettes (measured by abrasion resistance in accordance with the normative guidelines for this type of product). An interesting conclusion, however, is that while increasing the compaction pressure had a clearly positive effect on the density of the briquettes, it had an ambiguous effect on their durability [30]. This work is a valuable source of information on the possibilities of managing plastic waste in combination with wood sawdust, and above all on the properties of briquettes obtained from these materials.

Orisaleye et al. studied the effect of compaction parameters on the properties of briquettes obtained from abura tree sawdust. The researchers determined the correlation between briquetting temperature, compaction pressure, pressure holding time, and the compressive strength, durability and density of the briquettes obtained. To this end, based on the results of experimental studies, they used ANOVA analysis to determine the correlation between the individual variables. In the conclusions of the study, the authors pointed to the significant impact of compaction pressure on the strength and durability of the briquette, as well as the significant impact of all the tested variable parameters (pressure, temperature, pressure holding time) on the density of the briquette [31,32,33]. In addition to the obvious conclusions regarding the tested material, this study also indicates the possibility of using statistical tools to assess the impact of individual input parameters on the physical properties of briquettes.

The current state of knowledge on the processes of biomass densification in the form of sawdust is based on numerous scientific studies in which researchers most often present the results of studies correlating various input parameters of the briquetting process and the properties of sawdust with the physical properties of the briquettes obtained. Researchers most often focus on testing the strength and durability of briquettes as a function of the pressure and temperature of sawdust pressing. The analyses are performed using various tools, including statistical tools such as ANOVA. However, there is a lack of information in the literature on oak sawdust of different particle sizes, as well as on the correlation between briquette stiffness and variable process parameters.

The analysis of the above-mentioned scientific studies and their results prompted research into the process of compacting waste material in the form of oak sawdust as a post-production material, which, by imparting appropriate mechanical properties during the compaction process, can be used as a biofuel for energy purposes. The following presents research on the process of compacting oak sawdust, where the input data for the process were variable parameters such as particle size, moisture content, compaction force, and process temperature. The desired outcomes of the process are briquette density and Young’s modulus, as these indirectly characterize the calorific value and resistance to mechanical damage occurring during transport and storage. The novelty of the article is the proposal to use post-production waste from parquet flooring in the form of oak sawdust as a biofuel and the search, based on an already performed analysis of interactions (analysis of variance—ANOVA) between the input parameters of the process and the responses of the compaction process settings to make the process as energy-efficient as possible. This is precisely presented by the objective functions in the optimization of the selection of the input parameters of the process.

## 2. Materials and Methods

The material used in the research was oak sawdust obtained as a by-product of oak parquet flooring manufactured at a production plant in Greater Poland. The sawdust was seasoned for one year in a closed, dry room at room temperature.

**Figure 1:**
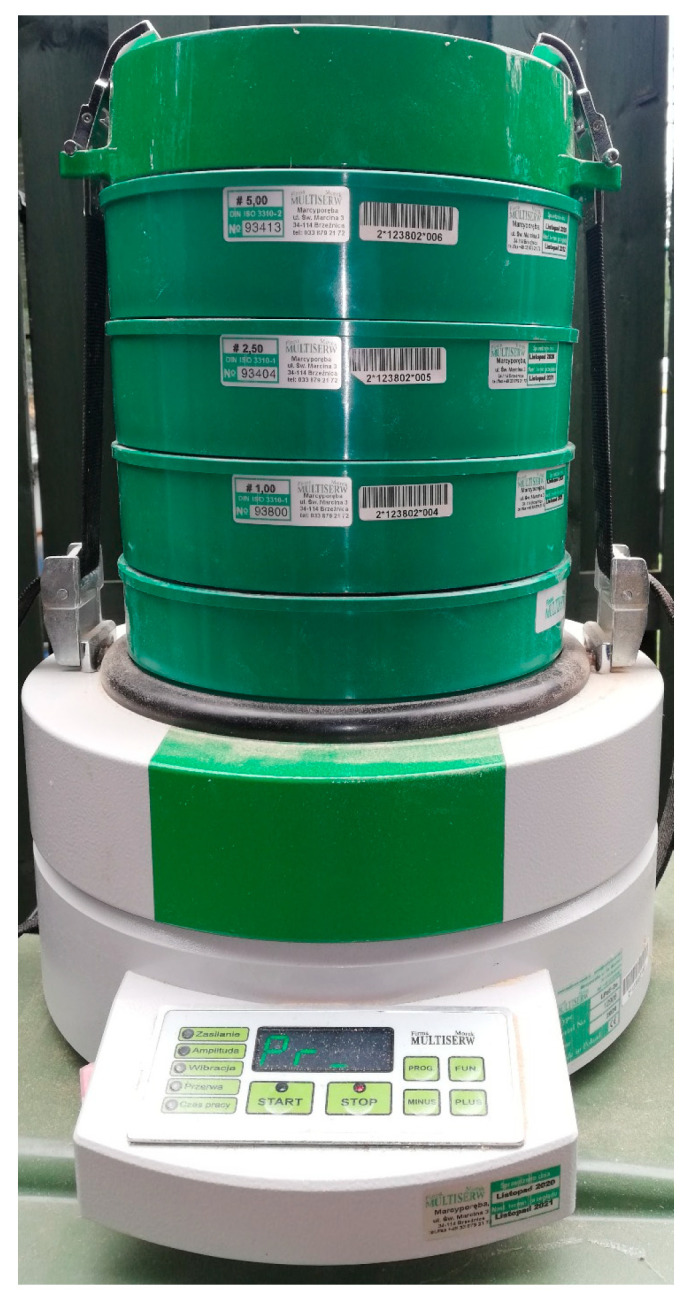
General view of laboratory sieve shaker (model LPzE-2e, manufactured by Multiserw-Morek, Brzeźnica, Poland).

The study used sawdust with two moisture levels. The first moisture level was approximately 9%, and sawdust with this moisture content was classified as dry. It was obtained by drying with a fan heater. This therefore requires additional electricity consumption. The second moisture level was 20%, which the sawdust reached as a result of aging in a dry room at room temperature. During drying, the moisture level was monitored on an ongoing basis until the desired level was achieved, using a Mettler Toledo moisture analyzer (Mettler Toledo, type HR83; Mettler-Toledo AG, 2009, Urdorf, Switzerland). Before the start of the tests, the sawdust was screened to obtain the appropriate fractions related to the particle size, which were then subjected to a compaction process (see Figure 1). For this purpose, a laboratory sieve model LPzE-2e, manufactured by Multi-serw-Morek, Brzeźnica, Poland (see Figure 1), was used, which was constructed using laboratory sieves compliant with [34,35].

**Figure 2:**
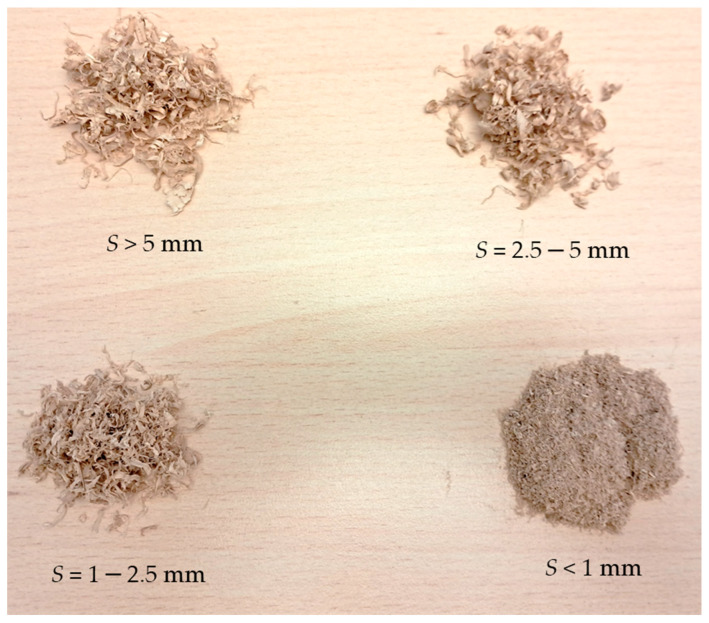
View of individual fractions of oak sawdust obtained in the sieving process.

This resulted in the following oak sawdust particle size fractions: *S* < 1 mm, *S* = 1–2.5 mm, *S =* 2.5–5 mm, *S* > 5 mm (see Figure 2).

**Figure 3:**
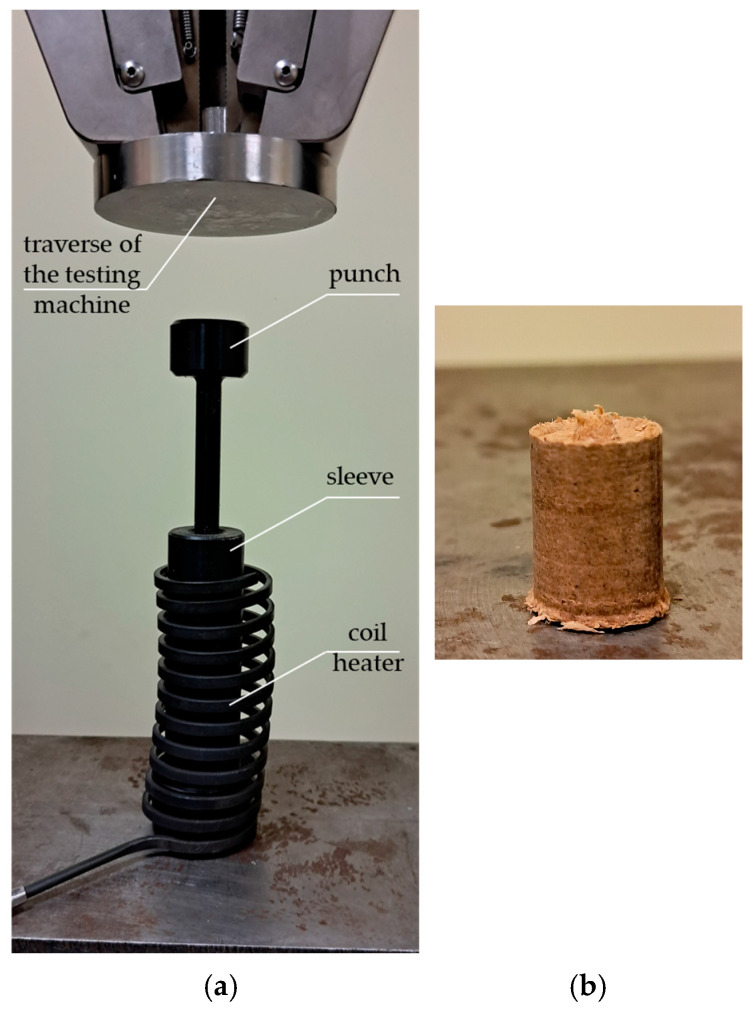
General view: (**a**) of the punch-sleeve assembly with the coil heater, (**b**) of the briquette obtained after the compaction test with a force of 25 kN at a temperature of approximately 25 °C.

The sawdust material prepared in this way was subjected to a compaction process with a force of 5 kN, 10 kN, 15 kN, 20 kN, and 25 kN at temperatures of 25, 100, 150, and 200 °C. Compaction was performed using a punch-bush assembly with a coil heater shown in Figure 3a. The diameter of both the punch and the sleeve was 10 mm.

For each set of process input parameters, i.e., temperature, compaction force, moisture content, and sawdust particle size, 10 compaction-unloading tests were performed. Based on the obtained characteristics of the change in compaction force (its increase to the set value) as a function of the displacement of the compaction piston, the density of the obtained briquette was determined. Before the test, the amount of sawdust that was poured into the sleeve and compacted by the punch, whose displacement and force were applied by the MTS Insight 50 kN strength testing machine (see Figure 3a), was weighed with a laboratory scale. After compaction, the volume of the briquette obtained was determined. The obtained information made it possible to determine the density *D* of the briquette produced in each test. Figure 3b shows an example of a briquette obtained from the experiments.

**Figure 4:**
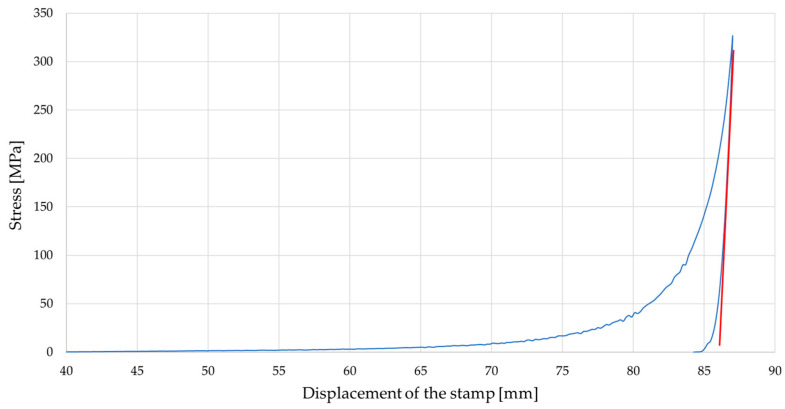
Characteristics of changes in compressive stress as a function of punch displacement for a compaction force of *F* = 25 kN, *T* = 25 °C and *S* < 1 mm.

One of the basic parameters required for numerical modeling using the Finite Element Method (FEM) is Young’s modulus. During the analysis of the compaction process, determining this parameter is challenging, as from the very beginning of the process a complex phenomenon occurs that simultaneously involves elastic and plastic deformations of the fragmented material particles. One of the commonly used methods for determining the averaged value of the elastic modulus for such materials is to perform a compaction test followed by unloading of the compacted sample [36]. Young’s modulus *E* for each conducted test (under specified settings) was determined based on the curve obtained from the compaction–unloading process. Specifically, the segment of the curve corresponding to the unloading phase was used. Two points were identified on this segment, through which a tangent to the unloading curve was drawn (see Figure 4, the red tangent line). Based on the stress and strain values determined at these points, the changes in stress and strain were calculated, which enabled the determination of Young’s modulus using the fundamental relationship:

*E* = *Δ*Stress/*Δ*Strain = (Stress_2_ − Stress_1_)/(Strain_2_ − Strain_1_) (1)

Figure 4 shows an example of the characteristic of changes in the compaction stress *σ_c_* as a function of the displacement of the piston, obtained on the basis of the tests carried out. The characteristic shown was performed for each test and was also used to determine the value of Young’s modulus.

Table 1 presents the average values of density *D* and Young’s modulus *E* from all samples.

## 3. Results and Discussion

In Table 1, the average values of the density *D* and Young modulus *E* for all the conditions applied during the sawdust densification process are presented. It should be remembered that for each process setting value the test was repeated 10 times.

The above research results were used for an Analysis of Variance (ANOVA). Table 2 presents the input data and the responses used for the analysis.

### 3.1. Multivariate Analysis of Density D

**Figure 5:**
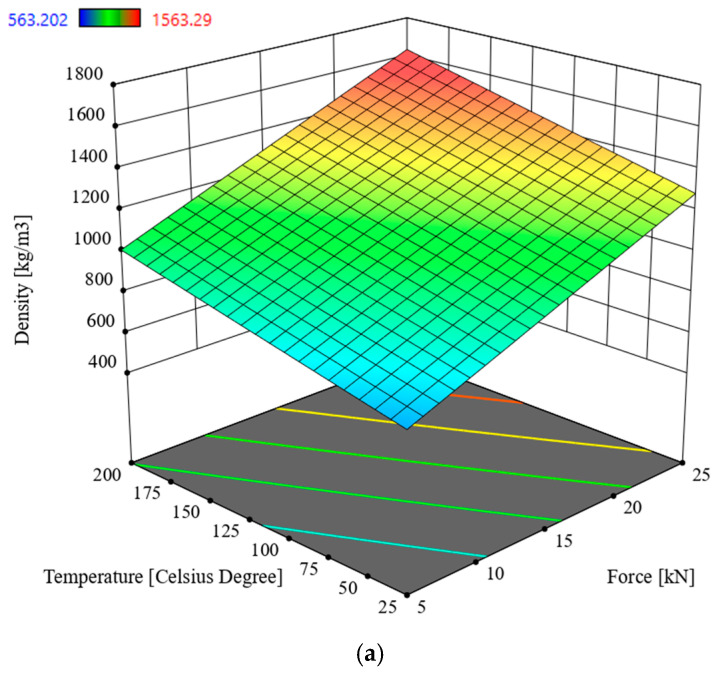

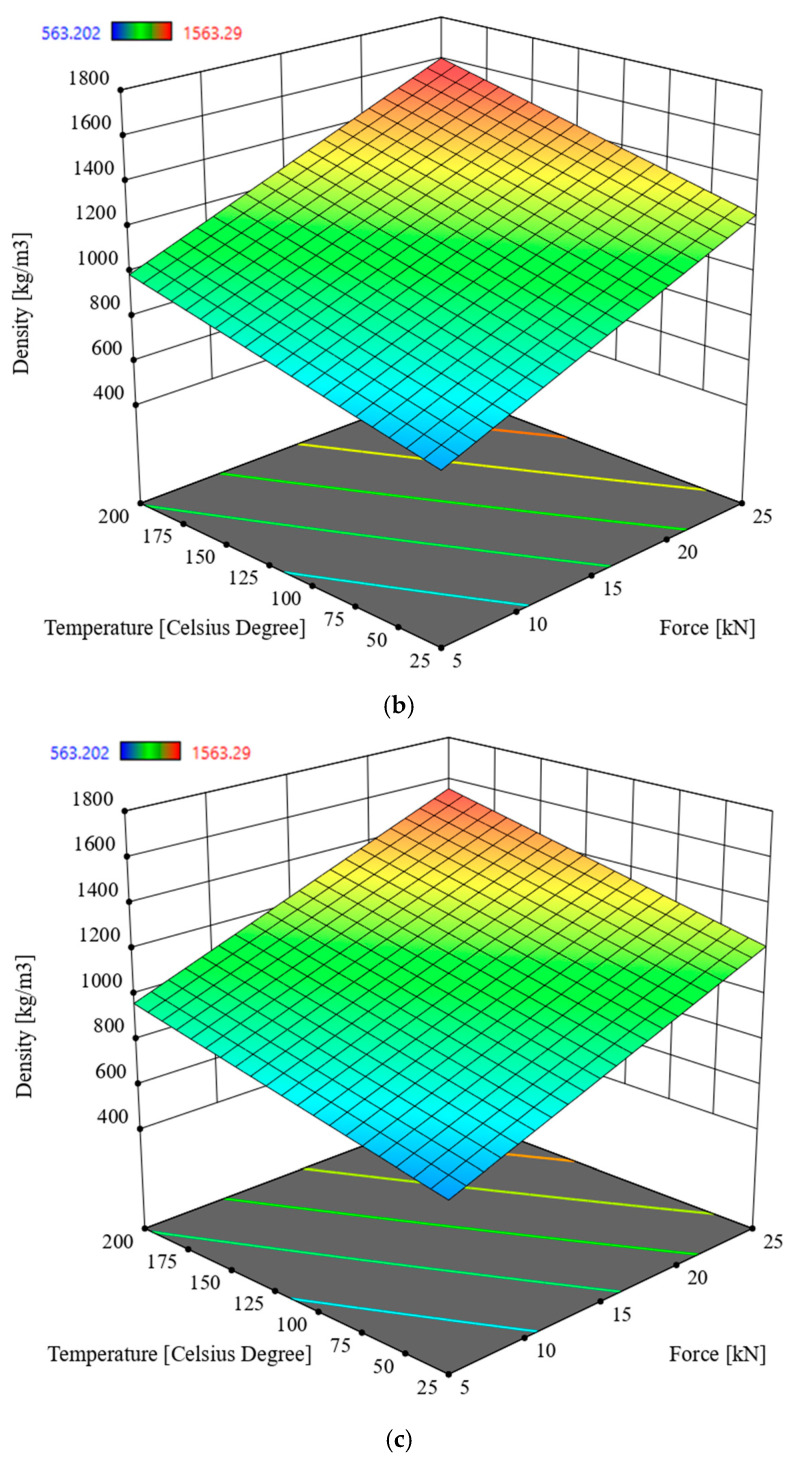

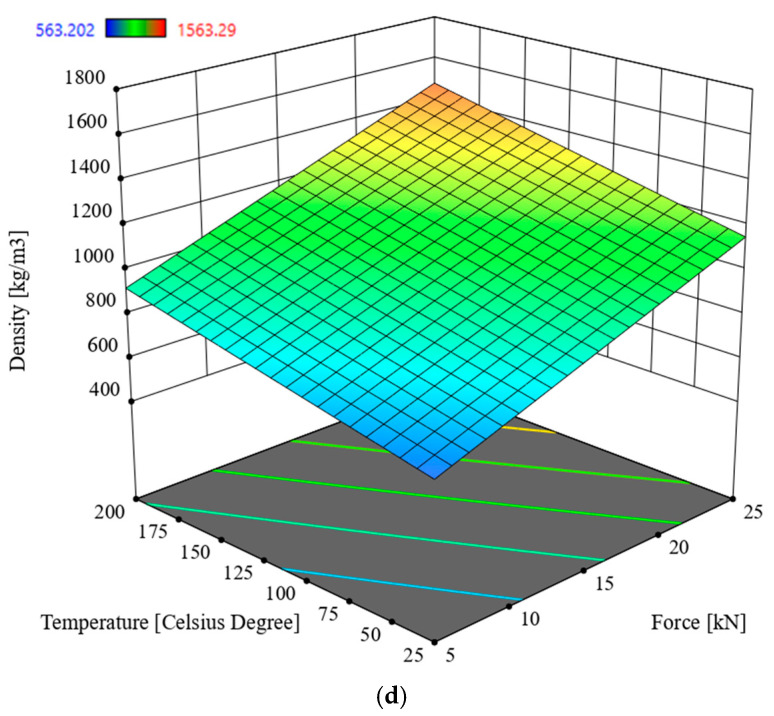
Density of oak sawdust briquettes, *D* in the function of the compaction force *F* and the temperature *T* for moisture value 9%: (**a**) particle size *S* < 1 mm, (**b**) *S =* 1–2.5 mm, (**c**) *S* = 2.5–5 mm, (**d**) *S* > 5 mm.

**Figure 6:**
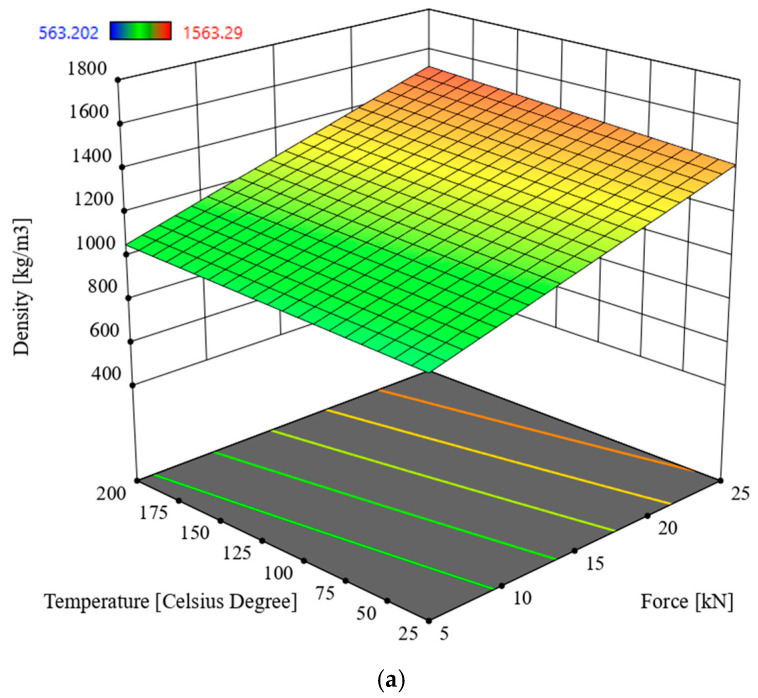

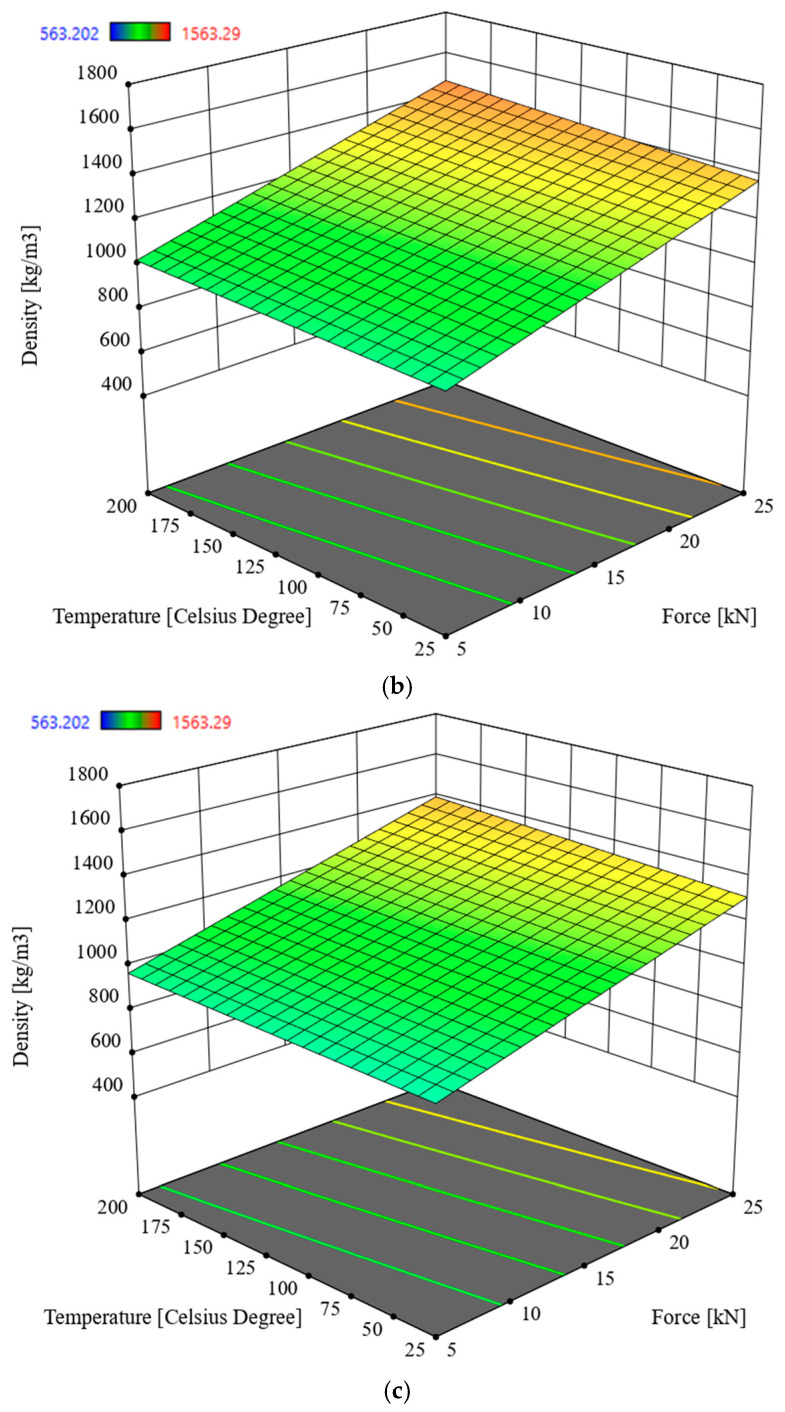

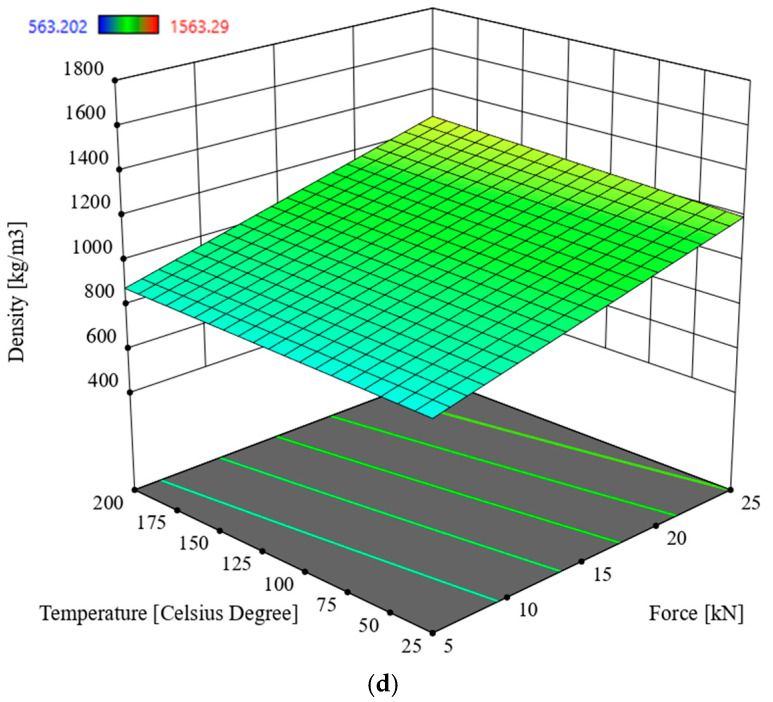
Density of oak sawdust briquettes, *D* in the function of the compaction force *F* and the temperature *T* for moisture value 20%: (**a**) particle size *S* < 1 mm, (**b**) *S* = 1–2.5 mm, (**c**) *S* = 2.5–5 mm, (**d**) *S* > 5 mm.

In order to determine the interactions between the input parameters of the process and the response in the form density *D* of oak sawdust briquettes, Analysis of Variance (ANOVA) was used. The analysis utilized the Reduced 2FI model, which is significant for the analyzed parameters and response. This model is characterized by a significance level of *p* < 0.0001 and an R^2^ = 0.8580. The model’s F-statistic value is 90.03 (see Table 3), indicating that the model is significant. The individual components of the model have a significance level *p* lower than 0.05, which makes the model significant. By using the model described by the correlation (2), it is possible to navigate the experimental design space, as the condition is met: (Predicted R^2^—Adjusted R^2^) < 0.2 and Adeg Precision = 43.6013. The model graph is shown in Figure 5 and Figure 6.

*D* = 192.93451 + 4.83688 × *S* + 18.02246 × *F +* 2.96192 *T +* 34.46923 × *M* − 0.323901 × *S* × *F −* 0.025348 × *S* × *T* − 1.61500 × *S* × *M* + 0.004380 × *F* × *T* − 0.372804 × *F* × *M* − 0.132079 × *T* × *M* (2)

where *D*—density [kg/m^3^], *S—*particle size [mm], *F*—compaction force [kN], *T*—temperature [°C], *M—*moisture [%].

Based on the data in Table 3, the parameter in the form of compaction force F has a significant impact on briquette density, followed hierarchically by temperature and particle size. The highest density value was recorded for the lowest particle size, the highest compaction force and compaction temperature values, and a moisture content of 9%.

### 3.2. Multivariate Analysis of Young Modulus E

**Figure 7:**
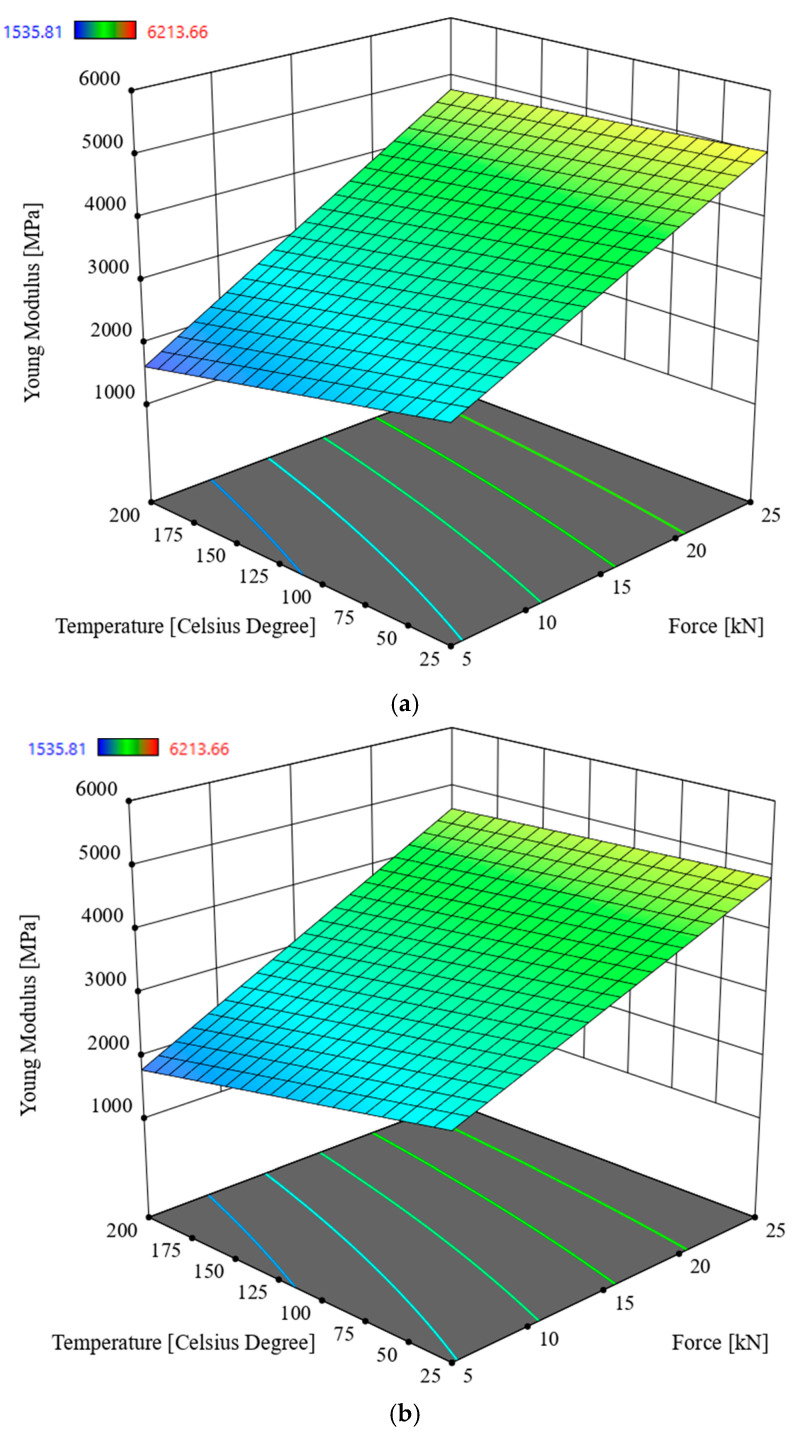

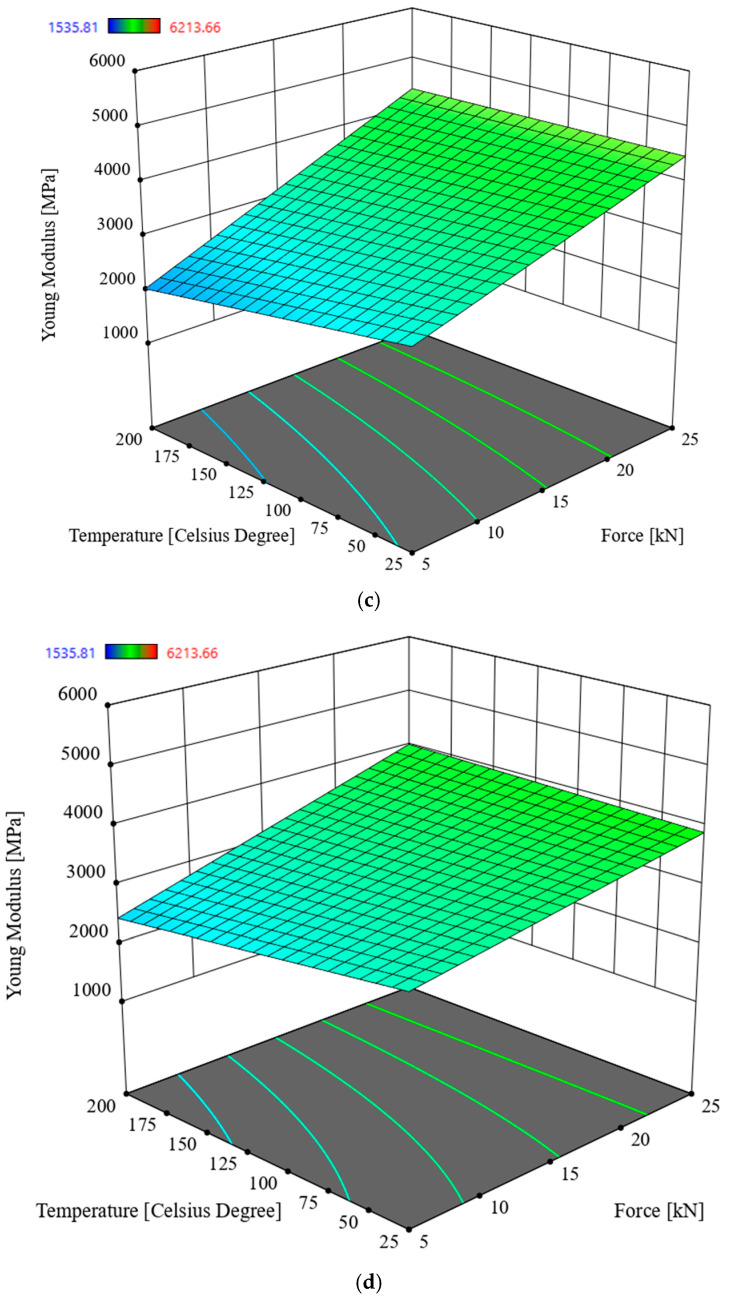
Young modulus *E* of oak sawdust briquettes, in the function of the compaction force *F* and the temperature *T* for moisture value 9%: (**a**) particle size *S* < 1 mm, (**b**) *S =* 1–2.5 mm, (**c**) *S =* 2.5–5 mm, (**d**) *S* > 5 mm.

**Figure 8:**
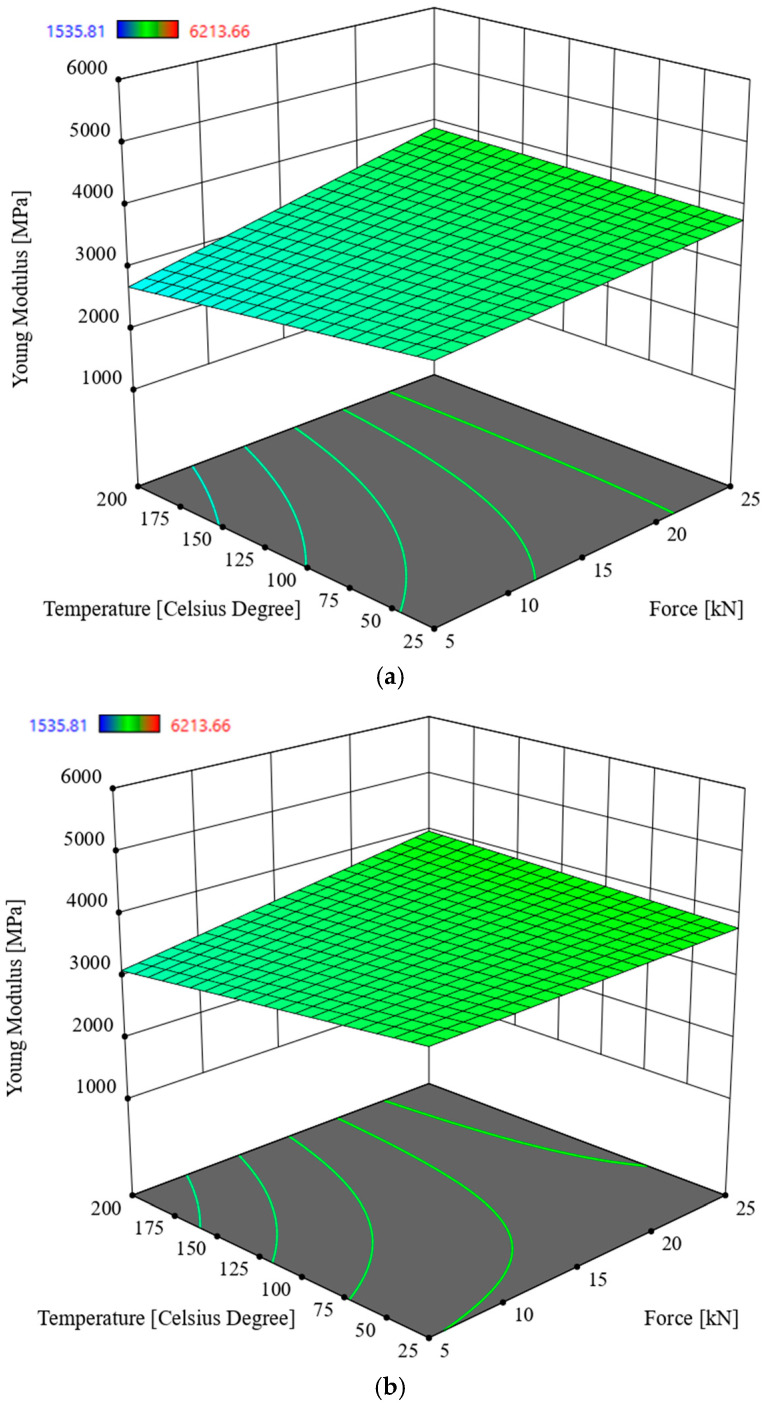

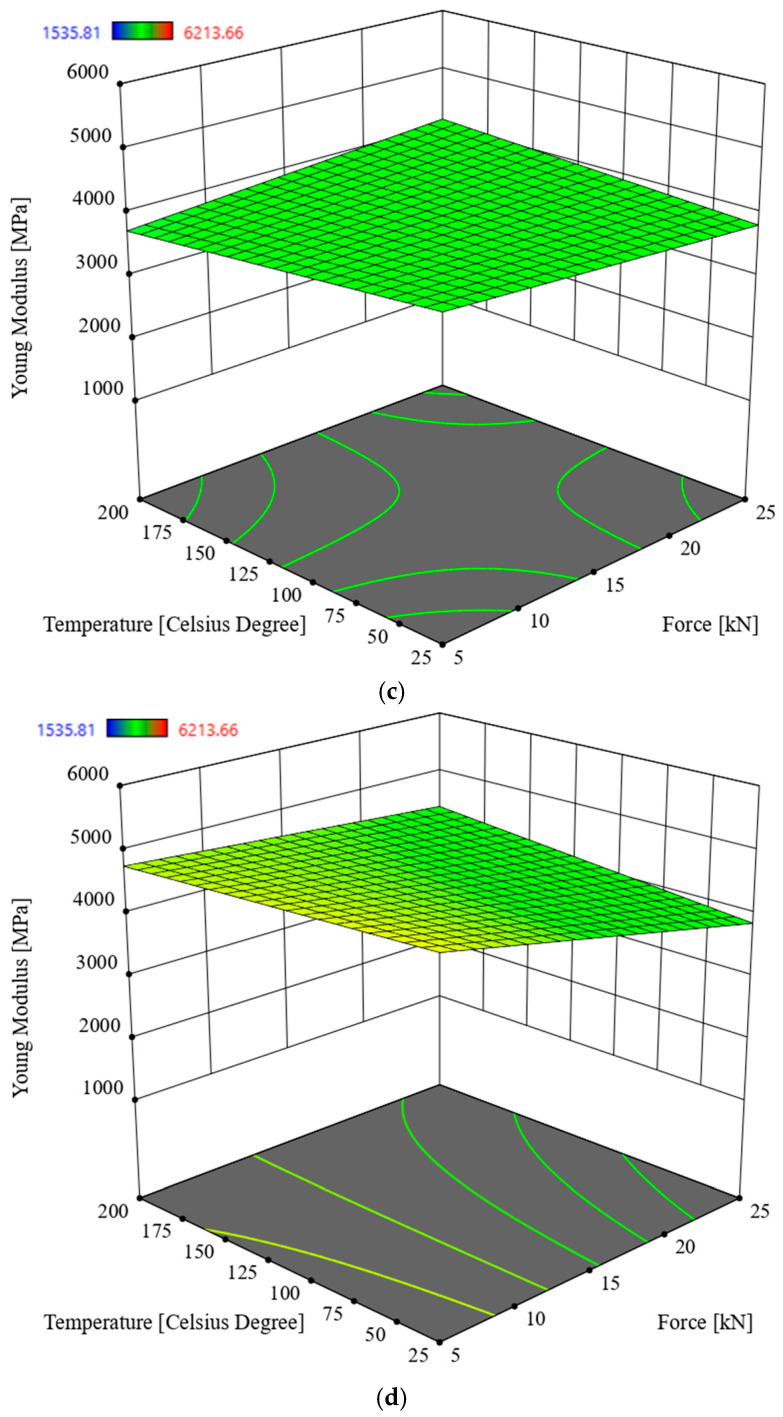
Young modulus *E* of oak sawdust briquettes in the function of the compaction force *F* and the temperature *T* for moisture value 20%: (**a**) particle size *S* < 1 mm, (**b**) *S =* 1–2.5 mm, (**c**) *S =* 2.5–5 mm, (**d**) *S* > 5 mm.

In order to determine the interactions between the input parameters of the process and the response in the Young modulus *E* of oak sawdust briquettes, Analysis of Variance (ANOVA) was used. The analysis utilized the Reduced 2FI model, which is significant for the analyzed parameters and response. This model is characterized by a significance level of *p* < 0.0001 and an R^2^ = 0.3221. The model’s F-statistic value is 7.08 (see Table 4), indicating that the model is significant. The individual components of the model have a significance level *p* lower than 0.05, which makes the model significant. By using the model described by the correlation (3), it is possible to navigate the experimental design space, as the condition is met: (Predicted R^2^—Adjusted R^2^) < 0.2 and Adeg Precision = 12.4208. The model graph is shown in Figure 7 and Figure 8.

*E* = 1360.61432 − 52.98012 × *S* + 193.06188 × *F −* 9.05474 × *T +* 101.41014 × *M* − 15.65047 × *S* × *F +* 0.472220 × *S* × *T* + 22.43142 × *S* × *M* + 0.221487 × *F* × *T* − 8.88581 × *F* × *M* + 0.207498 × *T* × *M* (3)

where *D*—density [kg/m^3^], *S—*particle size [mm], *F*—compaction force [kN], *T*—temperature [°C], *M—*moisture [%].

Based on the data contained in Table 4, the parameter in the form of compaction force *F* has the most significant impact on briquette density, followed hierarchically by moisture content, particle size, and temperature. The highest value of Young’s modulus *E* was recorded for 9% moisture content, the highest compaction force value, i.e., 25 kN, the lowest temperature value, and the lowest particle size (see Figure 7 and Figure 8).

### 3.3. Optimization of the Selection of Compaction Process Parameters

Based on the obtained test results and the determined values of density *D* and Young’s modulus *E*, the process parameters were optimized. The optimization criteria are presented in Table 5. Due to the desire to minimize the energy consumption of the compaction process, the criterion for the compaction force and compaction temperature values was to minimize these values. For the density *D* of the briquette and Young’s modulus *E*, the aim was to achieve their maximum values in order to obtain the best calorific value, which goes hand in hand with an increase in density, and to obtain the highest value of modulus *E* in order to achieve the best mechanical properties (durability), taking into account the storage and transport of the briquette.

The optimization results are presented in Table 6.

According to Table 6, the best input parameters for the process to meet the objectives set out in Table 5 will be to carry out the briquetting process of oak sawdust with particles in the range *S* = 2.5–5 mm, with a compaction force of approximately 5 kN at ambient temperature for sawdust moisture content of approximately 20%. This value of moisture content that the sawdust reaches after an appropriate period of aging in a room at room temperature, which does not require additional energy input for drying, except for the appropriate storage time, the amount of which will depend on the initial moisture content of the sawdust. The disadvantage of using a defined fraction, i.e., *S* = 2.5–5 mm, for compaction is the need to consume energy to power the sifter. Results of optimization show that, if we still insist on minimizing the energy required to produce briquettes, we can skip the sawdust sieving process, obtaining (see Figure 7 and Figure 8) briquettes with a density *D* that is about 3% lower and a Young’s modulus *E* that is approximately 6% higher, while maintaining the values of the other input parameters of the compaction process unchanged (see Table 6).

According to the analysis, the compaction force plays a key role, while the process temperature and sawdust moisture content also play a significant, albeit secondary, role. Carrying out the compaction process at a higher temperature is pointless, as it prolongs the material’s heating time and adversely affects its durability. Sitzmann and Buschhart also reached this conclusion [37]. The impact of the compaction force promotes the spread of lignin within the particle structure, where it is transferred to adjacent particles, causing them to bond. This is especially true because the particles deform under the influence of the compaction force, and the distances between them decrease, eliminating empty spaces. Proteins and starch may perform a similar function, gelling individual particles together in the presence of water [38]. Soo min Lee et al. found that during the compaction process of larch and tulipwood sawdust, increasing temperature and moisture improves pellet durability, and lignin is a natural binder between particles [39]. Robert Samuelsson et al., in turn, found that conducted a study on the densification of Scots pine and spruce sawdust. The results showed that low moisture content and long storage time had a positive effect on the increase in density, energy consumption of the pelletizer, mechanical strength of the pellets, and their quality [40]. According to the study results, in the case of densification of oak sawdust, a higher moisture content resulted in a lower density value, which negatively translated into relaxation (the authors noted that relaxation after densification reached higher values). According to So min Lee [39], higher moisture content has a positive effect, and according to Robert Samuelsson et al. [40], a negative effect on the densification process, which depends on the type of material being densified. Similar results were obtained by Roberto García et al. during the pelleting of pine sawdust. The team found that adding small amounts of lignin-rich additives improved the natural binding properties, and that a total moisture content of 13% and the addition of 10% to 20% glycerol improved the densification properties and increased the fuel’s calorific value [41]. The increase in density with increasing temperature, and the resulting higher density for a moisture content of 9% in sawdust, can be explained by the fact that evaporating water does not increase the sample volume, which causes an increase in briquette density. The softening effect of temperature causes a decrease in Young’s modulus *E* (Figure 5, Figure 6, Figure 7 and Figure 8), making the sawdust more susceptible to the applied densification force, which in turn contributes to an increase in briquette density [38]. Stefan Frodeson et al., who studied the effect of moisture content of compacted material from pine, spruce, and beech sawdust on the energy demand of the compaction process, found that when compacting with a low force of up to 5 kN, the water content of the material did not affect the amount of energy required for the compaction process. The situation was different for compaction with a force above this value. Another conclusion of the researchers was that moisture content has a significant effect on polysaccharide biomass, which is reflected in the compaction process [42]. In the present study, compacting sawdust with a force of 5 kN corresponds to a compressive stress of approximately 63.7 MPa for a sample compacted in a 10 mm diameter sleeve.

## 4. Conclusions

Research was conducted on the compaction process of waste material in the form of oak sawdust, for moisture content of 9% and 20%, compaction force *F* of 5, 10, 15, 20, and 25 kN, process temperatures of 25 °C, 50 °C, 100 °C, 150 °C, and 200 °C, and fractions obtained by screening with particle sizes in the ranges *S* < 1 mm, *S =* 1–2.5 mm, *S =* 2.5–5 mm, and *S* > 5 mm as process settings made it possible to determine the response in the form of the density *D* of the obtained briquette and its Young’s modulus *E*. ANOVA was used to analyze the interaction between the above-mentioned input parameters and the obtained responses. On this basis, the following conclusions were drawn and the selection of compaction process parameters was optimized:

- for sawdust moisture content of 9%, the most significant influence on briquette density *D* is exerted by compaction force *F*, followed by temperature *T* and particle size *S*,
- the highest density value was recorded for the lowest particle size, the highest compaction force and compaction temperature, and a moisture content of 9% (see Figure 5 and Figure 6),
- for sawdust moisture content of 20%, the parameter in the form of compaction force *F* has a significant impact on briquette density, followed by moisture content, temperature, and particle size,
- the highest Young’s modulus *E* was recorded for 9% moisture content, the highest compaction force of 25 kN, the lowest temperature, and the lowest particle size (see Figure 7 and Figure 8),
- analysis of variance (ANOVA) enabled the optimal selection of compaction process parameters, where the main criterion in general terms was to minimize the energy consumption of the compaction process,
- the best mechanical properties of the briquette, while maintaining the objective of minimizing the energy consumption of the process (see Table 5), can be obtained for the process settings of *F* = 5 kN, *M* = 20%, *T* = 25 °C, *S* = 2.5–5 mm (see Table 6),
- by foregoing sieving (i.e., further reducing the energy consumption of the process), we can obtain briquettes with a density *D* approximately 3% lower and a Young’s modulus *E* approximately 6% higher than that obtained from sawdust fractions with particle sizes *S* = 2.5–5 mm (this results by optimization was given where *F* = 5 kN, *M* = 20%, *T* = 25 °C, *S* > 5 mm, *D* = 840.4 kg/m^3^, *E* = 5016.6 MPa).

## Figures and Tables

**Table 1:** The averaged values of the experimental results from 10 trials are presented as responses to the specific input settings of the process.

Particle Size *S* [mm]	Compaction Force *F* [kN]	Compaction Temperature *T* [°C]	Moisture *M* [%]	Density *D* [kg/m^3^]	Standard Deviation of Density *σ_D_*	Young modulus *E* [MPa]	Standard Deviation of Young Modulus *σ_E_*
<1	5	25	9	625.8	31.3	2246.38	134.7
<1	5	100	9	996.7	69.8	1815.42	90.7
<1	5	150	9	958.2	76.7	1734.03	104.1
<1	5	200	9	975.7	19.5	1706.46	68.2
<1	10	25	9	719.6	14.4	3693.53	147.7
<1	10	100	9	1088.1	131.6	2893.23	318.2
<1	10	150	9	1052.9	128.4	2893.54	289.3
<1	10	200	9	1070.9	133.8	2951.51	354.1
<1	15	25	9	955.9	119.4	4491.69	539
<1	15	100	9	1317.3	165.9	3328.44	299.5
<1	15	150	9	1291.9	164.1	3738.39	299.1
<1	15	200	9	1311.8	167.9	3770.82	377.1
<1	20	25	9	1101.4	132.1	5335.04	533.5
<1	20	100	9	1456.8	180.6	4032.68	403.2
<1	20	150	9	1439.7	175.6	4247.47	509.6
<1	20	200	9	1461	179.7	4342.71	521.1
<1	25	25	9	1199.9	149.9	6213.66	745.6
<1	25	100	9	1549.4	195.2	5046.81	605.6
<1	25	150	9	1540.4	195.6	4965.78	595.8
<1	25	200	9	1563.3	190.7	4771.34	572.5
1–2.5	5	25	9	600.7	30.2	2134.06	128.2
1–2.5	5	100	9	956.8	66.9	1724.65	86.2
1–2.5	5	150	9	919.9	73.5	1647.33	98.8
1–2.5	5	200	9	936.6	18.7	1621.13	64.8
1–2.5	10	25	9	690.8	13.8	3508.85	140.3
1–2.5	10	100	9	1044.6	126.3	2748.57	302.3
1–2.5	10	150	9	1010.8	123.3	2748.86	274.8
1–2.5	10	200	9	1028.1	128.5	2803.93	336.4
1–2.5	15	25	9	917.7	114.7	4267.1	512.1
1–2.5	15	100	9	1264.6	159.3	3162.01	284.5
1–2.5	15	150	9	1240.3	157.5	3551.47	284.1
1–2.5	15	200	9	1259.4	161.1	3582.28	358.2
1–2.5	20	25	9	1057.4	126.8	5068.29	506.8
1–2.5	20	100	9	1398.5	173.4	3831.05	383.1
1–2.5	20	150	9	1382.1	168.6	4035.1	484.2
1–2.5	20	200	9	1402.6	172.5	4125.58	495.2
1–2.5	25	25	9	1151.9	143.9	5902.98	708.3
1–2.5	25	100	9	1487.4	187.4	4794.47	575.3
1–2.5	25	150	9	1478.8	187.8	4717.49	566.1
1–2.5	25	200	9	1500.8	183.1	4532.77	543.9
2.5–5	5	25	9	581.9	29.3	2089.14	125.3
2.5–5	5	100	9	926.9	64.8	1688.34	84.4
2.5–5	5	150	9	891.1	71.2	1612.65	96.7
2.5–5	5	200	9	907.4	18.1	1587.01	63.4
2.5–5	10	25	9	669.2	13.3	3434.98	137.3
2.5–5	10	100	9	1011.9	122.4	2246.38	295.9
2.5–5	10	150	9	979.2	119.4	1815.42	269.2
2.5–5	10	200	9	995.9	124.4	1734.03	229.3
2.5–5	15	25	9	889.1	111.1	1706.46	301.2
2.5–5	15	100	9	1225.1	154.3	3693.53	278.5
2.5–5	15	150	9	1201.5	152.5	2893.23	278.1
2.5–5	15	200	9	1220	156.1	2893.54	350.6
2.5–5	20	25	9	1024.3	122.9	2951.51	396.1
2.5–5	20	100	9	1354.8	167.9	4491.69	375.1
2.5–5	20	150	9	1338.9	163.3	3328.44	374.1
2.5–5	20	200	9	1358.7	167.1	3738.39	384.6
2.5–5	25	25	9	1115.9	139.4	3770.82	493.
2.5–5	25	100	9	1440.9	181.5	5335.04	563.2
2.5–5	25	150	9	1432.6	181.9	4032.68	554.1
2.5–5	25	200	9	1453.9	177.3	4247.47	532.4
>5	5	25	9	563.2	28.1	4342.71	221.3
>5	5	100	9	897	62.7	6213.66	581.6
>5	5	150	9	862.4	68.9	5046.81	493.6
>5	5	200	9	878.1	17.5	4965.78	461.4
>5	10	25	9	647.6	12.9	4771.34	332.9
>5	10	100	9	979.3	118.4	2134.06	286.4
>5	10	150	9	947.6	115.6	1724.65	260.4
>5	10	200	9	963.9	120.4	1647.33	318.7
>5	15	25	9	860.4	107.5	1621.13	185.1
>5	15	100	9	1185.6	149.3	3508.85	269.6
>5	15	150	9	1162.8	147.6	2748.57	269.1
>5	15	200	9	1180.7	151.1	2748.86	339.3
>5	20	25	9	991.3	118.9	2803.93	480.1
>5	20	100	9	1311.1	162.5	4267.1	362.9
>5	20	150	9	1295.7	158.4	3162.01	458.7
>5	20	200	9	1314.9	161.7	3551.47	469.1
>5	25	25	9	1079.9	134.9	3582.28	671.9
>5	25	100	9	1394.4	175.6	5068.29	545.5
>5	25	150	9	1386.4	176.1	3831.05	536.3
>5	25	200	9	1406.9	171.6	4035.1	515.3
<1	5	25	20	911.7	45.5	4125.58	207.6
<1	5	100	20	1211.8	84.8	5902.98	119.6
<1	5	150	20	1134.4	90.7	4794.47	144.7
<1	5	200	20	964.3	19.2	4717.49	102.3
<1	10	25	20	995.7	19.9	4532.77	166.2
<1	10	100	20	1269.3	153.5	2089.14	408.7
<1	10	150	20	1186.4	144.7	1688.34	398.7
<1	10	200	20	1056.5	132.5	1612.65	332.3
<1	15	25	20	1203	150.3	1587.01	228.2
<1	15	100	20	1408.4	177.4	3434.98	416.7
<1	15	150	20	1320.2	167.6	2690.71	328.5
<1	15	200	20	1288.6	164.9	2690.99	397.7
<1	20	25	20	1324.8	158.9	2744.9	263.9
<1	20	100	20	1486	184.2	4177.27	496.9
<1	20	150	20	1406.2	171.5	3095.44	467.2
<1	20	200	20	1431.1	176.1	3476.7	242.3
<1	25	25	20	1400.1	175.1	3506.86	301.9
<1	25	100	20	1528.9	192.6	4961.59	499.7
<1	25	150	20	1468.9	186.5	3750.39	227.5
<1	25	200	20	1527.1	186.3	3950.15	448.4
1–2.5	5	25	20	863.2	43.1	4038.72	197.2
1–2.5	5	100	20	1147.3	80.3	5778.71	113.6
1–2.5	5	150	20	1074	85.9	4693.53	137.5
1–2.5	5	200	20	912.9	18.2	4618.18	97.2
1–2.5	10	25	20	942.7	18.8	4437.34	157.9
1–2.5	10	100	20	1201.7	145.4	2021.74	388.3
1–2.5	10	150	20	1123.3	137.3	1633.88	378.8
1–2.5	10	200	20	1000.3	125.4	1560.63	105.7
1–2.5	15	25	20	1139	142.3	1535.81	196.8
1–2.5	15	100	20	1333.5	168.1	3324.18	295.9
1–2.5	15	150	20	1249.9	158.7	2603.91	207.1
1–2.5	15	200	20	1220.1	156.1	2604.18	272.8
1–2.5	20	25	20	1254.3	150.5	2656.36	235.7
1–2.5	20	100	20	1406.9	174.4	4042.52	472.1
1–2.5	20	150	20	1331.4	162.4	2995.59	333.8
1–2.5	20	200	20	1354.9	166.6	3364.55	210.2
1–2.5	25	25	20	1325.7	165.7	3393.74	266.8
1–2.5	25	100	20	1447.6	182.3	4801.54	569.7
1–2.5	25	150	20	1390.8	176.6	3629.41	591.1
1–2.5	25	200	20	1445.9	176.3	3822.72	516.2
2.5–5	5	25	20	795.3	39.7	3908.44	293.1
2.5–5	5	100	20	1057.1	73.9	5592.3	211.2
2.5–5	5	150	20	989.6	79.1	4542.13	134.6
2.5–5	5	200	20	841.2	16.8	4469.21	195.1
2.5–5	10	25	20	868.6	17.3	4294.2	154.6
2.5–5	10	100	20	1107.2	133.9	3460.73	380.1
2.5–5	10	150	20	1034.9	126.2	2393.14	370.8
2.5–5	10	200	20	921.6	115.2	2412.71	495.1
2.5–5	15	25	20	1049.4	131.1	2558.51	584.3
2.5–5	15	100	20	1228.6	154.8	4156.29	387.6
2.5–5	15	150	20	1151.7	146.2	3715.98	398.5
2.5–5	15	200	20	1124.1	143.8	3987.62	462.9
2.5–5	20	25	20	1155.6	138.6	4436.46	524.4
2.5–5	20	100	20	1296.3	160.7	5235.83	462.1
2.5–5	20	150	20	1226.7	149.6	4631.09	620.5
2.5–5	20	200	20	1248.4	153.5	5357.4	597.3
2.5–5	25	25	20	1221.4	152.6	4977.76	652.7
2.5–5	25	100	20	1333.7	168.2	5639.09	557.7
2.5–5	25	150	20	1281.4	162.7	4969.39	676.5
2.5–5	25	200	20	1332.1	162.5	5560.33	603.1
>5	5	25	20	766.2	38.3	5352.83	186.8
>5	5	100	20	1018.4	71.2	5849.41	107.6
>5	5	150	20	953.4	76.2	4998.18	130.2
>5	5	200	20	810.4	16.2	6062.66	92.1
>5	10	25	20	836.8	16.7	5404.1	149.6
>5	10	100	20	1066.7	129.2	3287.69	367.8
>5	10	150	20	997.1	121.6	2273.49	358.8
>5	10	200	20	887.9	110.9	2292.07	479.1
>5	15	25	20	1011	126.3	2430.58	465.4
>5	15	100	20	1183.6	149.1	3948.48	375.1
>5	15	150	20	1109.5	140.9	3530.18	385.7
>5	15	200	20	1082.9	138.6	3788.24	447.9
>5	20	25	20	1113.4	133.6	4214.64	407.5
>5	20	100	20	1248.9	154.8	4974.04	447.2
>5	20	150	20	1181.8	144.1	4399.54	600.5
>5	20	200	20	1202.7	147.9	5089.53	578.1
>5	25	25	20	1176.7	147.2	4728.87	631.7
>5	25	100	20	1284.9	161.9	5357.13	539.8
>5	25	150	20	1234.5	156.7	4720.92	654.7
>5	25	200	20	1283.4	156.5	5282.32	583.6

**Table 2:** A summary of the input coefficients and the responses used in the conducted ANOVA analysis.

Name	Units	Type	Low	High
Particle size, *S*	[mm]	Factor	<1	>5
Compaction force, *F*	[kN]	Factor	5	25
Temperature, *T*	[°C]	Factor	25	200
Moisture, *M*	[%]	Factor	9	20
Density, *D*	[kg/m^3^]	Response	563.2	1563.3
Young modulus, E	[MPa]	Response	1535.8	6213.7

**Table 3:** ANOVA results. Dependent variable—density—*D* [kg/m^3^].

Source	Sum of Squares	df ^a^	Mean Square	F-Value	*p*-Value	
Model	6.993 × 10^6^	10	6.993 × 10^5^	90.03	<0.0001	significant
*S*	5.455 × 10^5^	1	5.455 × 10^5^	70.24	<0.0001	
*F*	4.615 × 10^6^	1	4.615 × 10^6^	594.16	<0.0001	
*T*	7.994 × 10^5^	1	7.994 × 10^5^	102.92	<0.0001	
*M*	88,870.83	1	88,870.83	11.44	0.0009	
*S*. *F*	11907.50	1	11,907.50	1.53	0.2176	
*S*. *T*	1524.06	1	1524.06	0.1962	0.6584	
*S*. *M*	44,775.03	1	44,775.03	5.76	0.0176	
*F*. *T*	2565.38	1	2565.38	0.3303	0.5664	
*F*. *M*	1.345 × 10^5^	1	1.345 × 10^5^	17.32	<0.0001	
*T*. *M*	3.529 × 10^5^	1	3.529 × 10^5^	45.43	<0.0001	

^a^ degrees of freedom.

**Table 4:** ANOVA results. Dependent variable—Young modulus—*E* [MPa].

Source	Sum of Squares	df ^a^	Mean Square	F-Value	*p*-Value	
Model	7.883 × 10^7^	10	7.883 × 10^6^	7.08	<0.0001	significant
*S*	4.620 × 10^6^	1	4.620 × 10^6^	4.15	0.0434	
*F*	1.907 × 10^7^	1	1.907 × 10^7^	17.13	<0.0001	
*T*	1.532 × 10^6^	1	1.532 × 10^6^	1.38	0.2427	
*M*	1.043 × 10^7^	1	1.043 × 10^7^	9.37	0.0026	
*S*. *F*	6.950 × 10^6^	1	6.950 × 10^6^	6.24	0.0136	
*S*. *T*	5.289 × 10^5^	1	5.289 × 10^5^	0.4750	0.4917	
*S*. *M*	8.638 × 10^6^	1	8.638 × 10^6^	7.76	0.0060	
*F. T*	1.640 × 10^6^	1	1.640 × 10^6^	1.47	0.2268	
*F. M*	1.911 × 10^7^	1	1.911 × 10^7^	17.16	<0.0001	
*T. M*	8.710 × 10^5^	1	8.710 × 10^5^	0.7823	0.3779	

^a^ degrees of freedom.

**Table 5:** Assumed optimization criteria.

Input/Output Variable	Goal	Lower Limit	Upper Limit
*S* [mm]	is in range	<1	>5
*F* [kN]	minimize	5	25
*T* [°C]	minimize	25	200
*M* [%]	is in range	9	20
*D* [kg/m^3^]	maximize	563.2	1563.3
*E* [MPa]	maximize	1535.8	6213.7

**Table 6:** Process parameters that meet the optimization criteria.

*S* [mm]	*F* [kN]	*T* [°C]	*M* [%]	*D* [kg/m^3^]	*E* [MPa]
2.5–5	5	25	20	867	4737.2

## Data Availability

The original contributions presented in this study are included in the article. Further inquiries can be directed to the corresponding author.
